# Soil environment reshapes microbiota of laboratory-maintained Collembola during host development

**DOI:** 10.1186/s40793-022-00411-7

**Published:** 2022-04-05

**Authors:** Duleepa Pathiraja, June Wee, Kijong Cho, In-Geol Choi

**Affiliations:** 1 Department of Biotechnology, College of Life Sciences and Biotechnology, Korea University, Seoul, 02841 Korea; 2 Department of Environmental Science and Ecological Engineering, College of Life Sciences and Biotechnology, Korea University, Seoul, 02841 Korea; 3 BK21 FOUR R&E Center for Environmental Science and Ecological Engineering, Korea University, Seoul, 02841 Korea

**Keywords:** Collembola, Invertebrates, Laboratory-maintained arthropod, Host–microbiota interactions, Environment–microbiota interactions

## Abstract

Collembola are soil-dwelling arthropods that play a key role in the soil ecosystem. *Allonychiurus kimi* (Lee) (Collembola: Onychiuridae) was isolated from the natural environment and has been maintained for 20 years under laboratory conditions. Though the morphological and physiological features of *A. kimi* are being widely used to evaluate the impact of pesticides and heavy metals on the soil ecosystem, variations observed in these features might be on account of its microbiota. However, the microbiota composition of the laboratory-maintained *A. kimi* is undetermined and how the community structure is changing in response to soil environments or interacting with the soil microbiota are still unknown. In this study, we determined the microbiota of laboratory-maintained *A. kimi* at both adult and juvenile stages and examined how the microbiota of *A. kimi* is affected by the microbial community in the soil environments. *Chryseobacterium*, *Pandoraea, Sphingomonas, Escherichia–Shigella,* and *Acinetobacter* were the core microbiota of *A. kimi.* Exposure of the laboratory-maintained *A. kimi* to different soil microbial communities drove dynamic shifts in the composition of *A. kimi* microbiota. Microbial association network analysis suggested that gut microbiota of lab-grown *A. kimi* was affected by exposing to soil microbial community. This study implies that shifts in the bacterial community of adult *A. kimi* can be utilized as an indicator to evaluate the soil ecosystem.

**Supplementary Information:**

The online version contains supplementary material available at 10.1186/s40793-022-00411-7.

## Introduction

Soil ecosystems are rich habitats for a wide range of microbial and fauna communities. Interactions between these two communities vary from simple predator–prey interactions to shaping the health and fertility of the soil ecosystem [23, 32]. The soil fauna plays a key role in the dynamics of the soil microbial community and soil biomass [10, 24]. Soil microbial and faunal communities together mediate soil formation, microclimate regulation, and disease control [14, 21, 33]. Although many of these functions are aided by the diverse microbial communities colonized in the soil fauna [17], the ecological role of soil inhabitants and their microbiota as a single unit is less well studied.

Collembola are highly abundant, soil-dwelling microarthropods, forming an important part of the soil fauna community [41]. Owing to their small size, diverse ecological preferences, and ease of sampling, Collembola serve as bioindicators of ecosystem health and soil quality [22]. Among the many Collembola species studied so far, the European parthenogenetic Collembola species, *Folsomia candida* is widely being used as an internationally standardized test species to monitor the soil quality [20]. Bacterial 16S rRNA and fungal ITS amplicon sequencing has produced a comprehensive overview on the microbiome of *F. candida* [1]. However, it has very little ecological relevance to soil ecosystems in the Korean peninsula, due to its low abundance [43]. *Allonychiurus kimi* (Lee) is a common Collembolan species native to Korean soils and is also known to be abundant in paddy fields of Korea [26]. *A. kimi* was listed as an alternative to *F. candida* for toxicity tests in Organization for Economic Co-operation and Development (OECD) 232 guidelines for testing chemicals [37] and it is used as an ecotoxicological test animal in accordance with the International Organization for Standardization (ISO) guidelines [45, 46]. The pure-bred model organism, *A. kimi*, has been maintained under constant laboratory conditions with controlled feeding for 20 years, ever since the species was isolated from a natural soil environment [26].

Several studies have been conducted on the environmental factors (such as temperature, humidity, nutrients, and soil contaminants), which affect the viability and reproduction of *A. kimi* [44–47]. The changes in individual features of *A. kimi* have widely been used for monitoring the soil quality in those studies. However, the composition of *A. kimi* microbiota and its interactions with the soil environment are largely elusive. Owing to constant rearing under laboratory conditions, *A. kimi* is likely to have a stable microbiota. We assume that the microbiota of lab‐grown *A. kimi* may change once it is exposed to different field soils. Thus, the microbiota of *A. kimi* can be used as a model system to assess the quality and the health of the soil ecosystem.

In the present study, we report a comprehensive overview of the bacterial community composition of a model Collembola, *A. kimi*, and lay the groundwork for future ecotoxicological studies supported by bacterial community data. We introduced a laboratory-maintained population of *A. kimi* into the soil with different physicochemical properties (e.g., sandy or clay loam) collected from two distinct geographical locations in the Republic of Korea. Temporal changes in the bacterial community structure of both the *A. kimi* and the soil were characterized using amplicon sequencing. Microbial interactions and putative keystone taxa were identified using the co-occurrence microbial interaction analysis. Further, by allowing the adult individuals to reproduce under the same conditions, we examined the microbiota of the first-generation juveniles and compared it with that of the adults. Thereby, we assessed the contribution of vertical transmission of the parental microbiota and acquisition of microbiota from the environment during the development of *A. kimi*.

## Materials and methods

### Test species and culture conditions.

*Allonychiurus kimi* (Lee) (formerly known as *Paronychiurus kimi*) was originally isolated from the paddy soil in the Republic of Korea and has been reared for 20 years under defined feeding conditions [26]. The organisms were grown on a moist substrate consisting of plaster of Paris, activated charcoal, and distilled water in plastic petri dishes (9.5 cm in diameter, 1.5 cm in height) that were filled up to ~ 0.5 cm of the height with the media. *Allonychiurus kimi* cultures were maintained at 20 ± 1 °C under continuous darkness and fed with Brewer’s yeast [26]. To obtain age synchronized adult *A. kimi* populations, 100 adults were separately introduced into several breeding substrates and allowed to lay eggs and then a cohort of eggs was transferred to a new substrate. After the eggs were hatched, the juveniles were cultured under the same conditions. A homogenous, age synchronized (42–46 days old) population of *A. kimi*, prepared using this procedure, was used in this study.

### Soil sampling and preparation

**Fig. 1:**
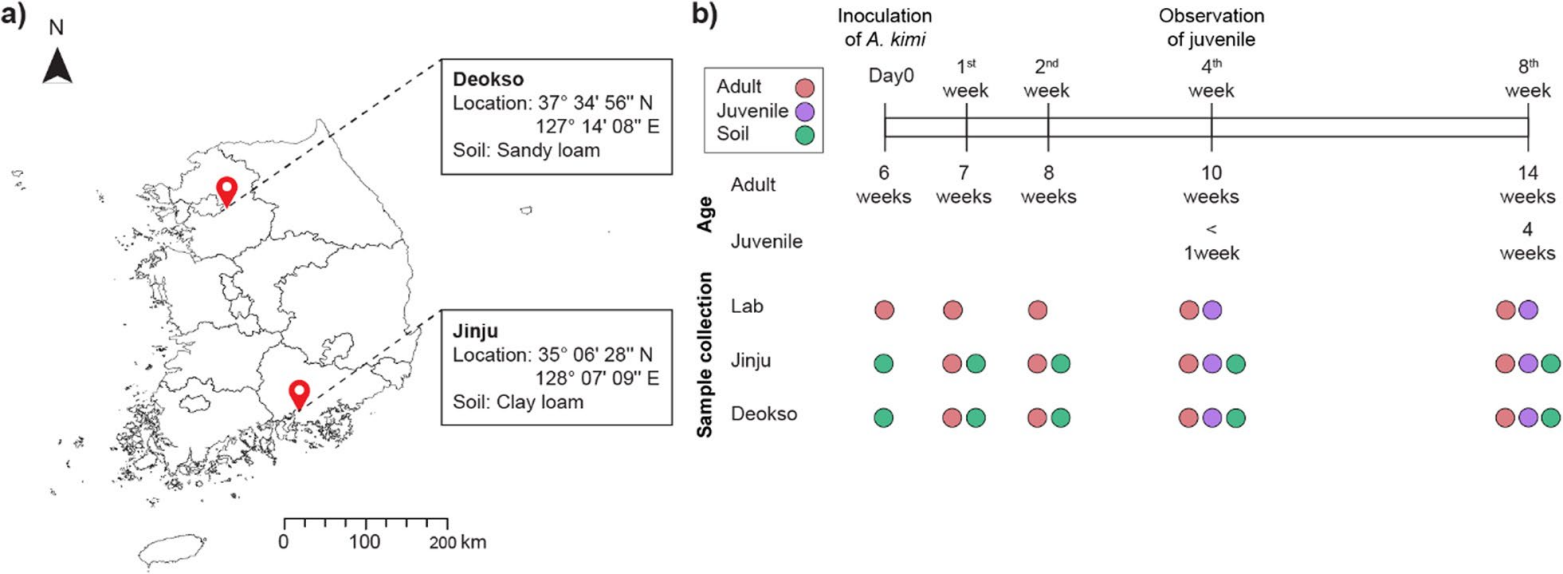
Overview of experimental design. **a** The soil collection sites, Deokso and Jinju are indicated on a map of the Republic of Korea. **b** Schematic representation of experimental design. Experiment was started with an age-synchronized homogenous population of adult *A. kimi.* Sampling time and age of adult and juvenile *A. kimi* and the type of the sample collected at each sampling time point are indicated

The soil used in this experiment was sampled at a depth of 10 cm from Deokso (N 37° 34′ 56″, E 127° 14′ 8″) and Jinju (N 35° 06′ 28″, E 128° 07′ 09″) in October 2017. The two sampling sites were approximately 290 km apart (Fig. 1a). In each sampling site, ten soil samples at a distance of at least 3 m apart were taken using a soil core sampler (diameter: 5 cm, height: 10 cm) at a depth of 10 cm except for the organic layer. The sampled soil was mixed thoroughly and was sieved through a 2 mm mesh to remove stones, debris, plant materials and large soil animals such as ground beetles of the Carabidae family. To preserve the soil microbial community while removing the soil fauna, the sieved soil was frozen at − 20 °C for 48 h and then allowed to be thawed for 24 h at room temperature in the dark. This procedure was repeated three times. This method has been used for defaunation with a minimal impact on the microbial community [39, 50]. For each sampling site, four polystyrene vessels (for four sampling times) each containing 100 g of defaunated soil were prepared immediately before inoculating *A. kimi.* An additional soil sample, which represents day0, from each site was stored at − 80 °C until DNA extraction.

### Experimental setup

On the day0, a homogenous population of 30 *A. kimi* adults (42–46 days old) were introduced into each vessel containing soil from two sampling sites, Deokso and Jinju. The test vessels were kept at 20 ± 1 °C under continuous darkness. The test vessels were aerated and weighed weekly to replenish the moisture loss by the addition of sterile deionized water, if needed. After 1, 2, 4, and 8 weeks, the surviving adults and juveniles produced in each vessel were floated with adding sterile deionized water. Adults and juveniles were separated based on their morphological differences [46] and were transferred into separate 2 ml tubes for DNA extraction. Juveniles were found only in sampling time points at 4 and 8 weeks. Additionally, 10 g of soil was collected from each vessel for DNA extraction. As the control group, a pool of 30 *A*. *kimi* adults (42–46 days old) were introduced into a fresh moist substrate used for culture. The moist substrates in the Petri dishes were maintained at 20 ± 1 °C in continuous darkness and provided with Brewer’s yeast as food weekly. After that, the surviving adults and juveniles were sampled at the same time points mentioned above. Additionally, we used the starting homogenous population of *A. kimi* as day zero. All samples were preserved at − 80 °C until DNA extraction. All together, we collected 13 adult *A. kimi* samples (3 culture conditions × 4 sampling times, and adults at day zero), 6 juvenile *A. kimi* samples (3 culture conditions × 2 sampling times), and 10 soil samples (2 culture conditions × 5 sampling times including day zero) for sequencing (Fig. 1b).

### DNA extraction from *A. kimi* and soil.

A pool of 30 adult individuals or 60 juvenile individuals was collected at each time point from each culture condition and used for the extraction of total DNA. Before the DNA extraction, *A. kimi* samples were washed twice for 2 min each with 70% ethanol and subsequently washed with sterile phosphate buffered saline, to remove the microorganisms attached to the body surface [18]. From each plate, 200 mg of soil was sampled and used as the starting material for DNA extraction. Total DNA of *A. kimi* and soil were extracted using the Quick DNA Fecal/Soil microbe mini prep kit (Zymo Research, Irvine, CA, USA), according to the manufacturer’s protocol.

### Bacterial 16S rRNA gene sequencing

Bacterial 16S rRNA gene sequencing was used to analyze the microbial community structure. All the primers were synthesized by Integrated DNA Technologies (IDT, Singapore). The V3–V4 regions of 16S rRNA gene were amplified using the following universal primer set (forward 5′-CCTACGGGNGGCWGCAG-3′, reverse 5′-GACTACHVGGGTATCTAATCC-3′) [29], and Q5 high fidelity DNA polymerase (NEB, Ipswich, MA, USA). Illumina overhang adapter sequences were added to the above universal primer set (forward overhang 5′-TCGTCGGCAGCGTCAGATGTGTATAAGAGACAG‐[locus specific sequence], reverse overhang: 5′-GTCTCGTGGGCTCGGAGATGTGTATAAGAGAC AG‐[locus specific sequence]. The thermocycler parameters were as follows: initial denaturation 3 min at 98 °C, followed by 20 amplification cycles (30 s at 98 °C, 30 s at 55 °C, 1 min at 72 °C), final extension 5 min at 72 °C. The resulting DNA amplicons were purified using the Agencourt AMPure XP beads (Beckman Coulter, Brea, CA, USA). A second PCR was performed to attach Illumina universal p5/p7 overhang sequences and sample specific barcodes (forward 5′-CAAGCAGAAGACGGCATACGAGAT[i7]GTCTCGTGGGCTCGG, reverse 5′-AATGATACGGCGACCACCGAGATCTACAC[i5]TCGTCGGCAGCGTC; where [i7] and [i5] are 6 base-pair sample specific barcodes) The thermocycler parameters for the second PCR were as follows: initial denaturation 1 min at 98 °C, followed by 12 amplification cycles (15 s at 98 °C, 15 s at 55 °C, 30 s at 72 °C), final extension 3 min at 72 °C. Amplification was performed using Q5 high fidelity DNA polymerase (NEB, Ipswich, MA, USA). Sequencing ready libraries were purified using the Agencourt AMPure XP beads (Beckman Coulter, Brea, CA, USA). Sequencing was conducted using the Illumina MiSeq Platform (Illumina, Diego, CA, USA) with MiSeq Reagent kit V3 (2 × 300 PE) (Illumina, Diego, CA, USA).

Control experiments for DNA extraction, library preparation and sequencing were conducted as follows. A mock DNA library was prepared with the ZymoBiomics microbial community DNA standard (Zymo Research, Irvine, CA, USA), using the amplicon library preparation procedure described above. This control library allowed us to assess the potential bias and errors associated with the amplification and sequencing steps. DNA extraction negative control was carried out by following the same protocol without soil or *A. kimi*. The negative control for PCR was carried out using the DNA extraction negative control as the template and following the same PCR conditions. We confirmed that the samples were not contaminated during DNA extraction or library preparation.

### Bioinformatic processing of sequencing data

Quality control of raw Illumina sequence reads was performed using Trim Galore ver. 0.5.0 (https://www.bioinformatics.babraham.ac.uk/projects/trim_galore/). Illumina universal adapter sequences were trimmed, reads shorter than 40 bp were removed and bases with Q < 20 were trimmed from the 3′ and 5′ ends of reads. Forward and reverse reads were merged into concatenated reads using PEAR ver. 0.9.8 [55]. The merged reads were clustered into Operational Taxonomic Units (OTUs) at 97% sequence similarity, using QIIME ver. 1.9.1 [13], which is an open-source bioinformatics pipeline for performing microbiome analysis. The SILVA database release (ver. 132) was used as the reference database for taxonomic assignment in the QIIME workflow [40]. Statistical analysis and visualization were performed using R version 3.6.0 [49] via RStudio version 1.2.1335 [48]. The R packages, “phyloseq” [36], “DEseq” [52] and “microbiome” [31] were used to analyze the diversity of the microbial community.

Alpha diversity was assessed based on the observed OTUs, Chao1, Shannon and Simpson diversity indices. As the alpha diversity indices did not meet the assumption of equal variance, the differences in alpha diversity between samples were calculated using the non-parametric Wilcoxon rank-sum test (Mann–Whitney). Beta diversity was analyzed using the Bray–Curtis dissimilarity and sample ordination was visualized using the multi-dimensional scaling (MDS). Permutational Multivariate Analysis of Variance (PERMANOVA) test was performed to test whether the sample groups differed significantly.

### Construction of ecological networks

OTUs with a relative abundance of at least 0.01% were used to construct a bacterial co-occurrence network for adult *A. kimi* using the SParse InversE Covariance estimation for Ecological Association Inference (SPIEC-EASI) method [30]. This method is robust to the challenges encountered when using compositional data with low sample counts. We then transformed the OTU count data, and network analysis was performed using the neighborhood selection (MB method) with a minimum lambda threshold of 0.01 for graphical model inference. All calculations were made using the SpiecEasi R package (version 1.0.7) [30]. We used the “igraph” R package [15] for generating the network plots. The network plots were then exported to Cytoscape (version 3.7.2) [42] for visualization. The ‘Network analyzer’ tool in Cytoscape was used to determine the network topology parameters. We used three parameters, (1) degree, (2) closeness centrality (3) betweenness centrality, to predict putative keystone taxa. Degree is defined as the number of edges connected to a node. Closeness centrality depends on the average shortest path and represents the central importance of a node. Betweenness centrality defines the role of a node as a bridge in the network. OTUs with highest degree, highest closeness of centrality and lowest betweenness centrality score were predicted as putative keystone taxa as defined in previous studies [8, 12]

## Results

### Physicochemical properties of the soil environments exposed to the laboratory-maintained *A. kimi*

To investigate the effects of soil environments for the microbiota of the laboratory-maintained *A. kimi*, we selected two geologically distant soil sampling sites in the Republic of Korea: Deokso and Jinju (Fig. 1a) and analyzed the physicochemical properties of the soils (Table 1). The soil sampled from Deokso was sandy loam, containing 64.5% sand, 16.7% slit, and 18.8% clay. It is classified as Fragiudults with a pH of 6.55 ± 0.02, and soil and water at a ratio of 1:5, w/w. The soil sampled in Jinju was clay loam containing 32.4% sand, 35.3% slit, and 32.3% clay, classified as Paleudults with a pH of 6.16 ± 0.09 and soil and water at a ratio of 1:5, w/w. Deokso and Jinju soils had similar organic matter contents, of 1.81% and 1.60%, respectively.

**Table 1:** Physico-chemical parameters of Deokso and Jinju soil

Sampling site	Deokso	Jinju
Location	37° 34′ 56″ N 127° 14′ 08″ E	35° 06′ 28″ N 128° 07′ 09″ E
Soil texture	Sandy loam (Fragiudults)	Clay loam (Paleudults)
Sand (%)	64.5	32.4
Silt (%)	16.7	35.3
Clay (%)	18.8	32.3
pH	6.55 ± 0.02	6.16 ± 0.09
Maximum WHC (g g^−1^ soil)	0.66 ± 0.01	0.86 ± 0.02
NH_4_^+^ N (mg kg^−1^)	2.52 ± 0.78	7.44 ± 2.31
NO_3_^−^ N (mg kg^−1^)	18.31 ± 6.04	7.71 ± 0.55
Organic matter (%)	1.81 ± 0.10	1.60 ± 0.13

### Overall bacterial community in the laboratory-maintained *A. kimi* and the soil samples

**Fig. 2:**
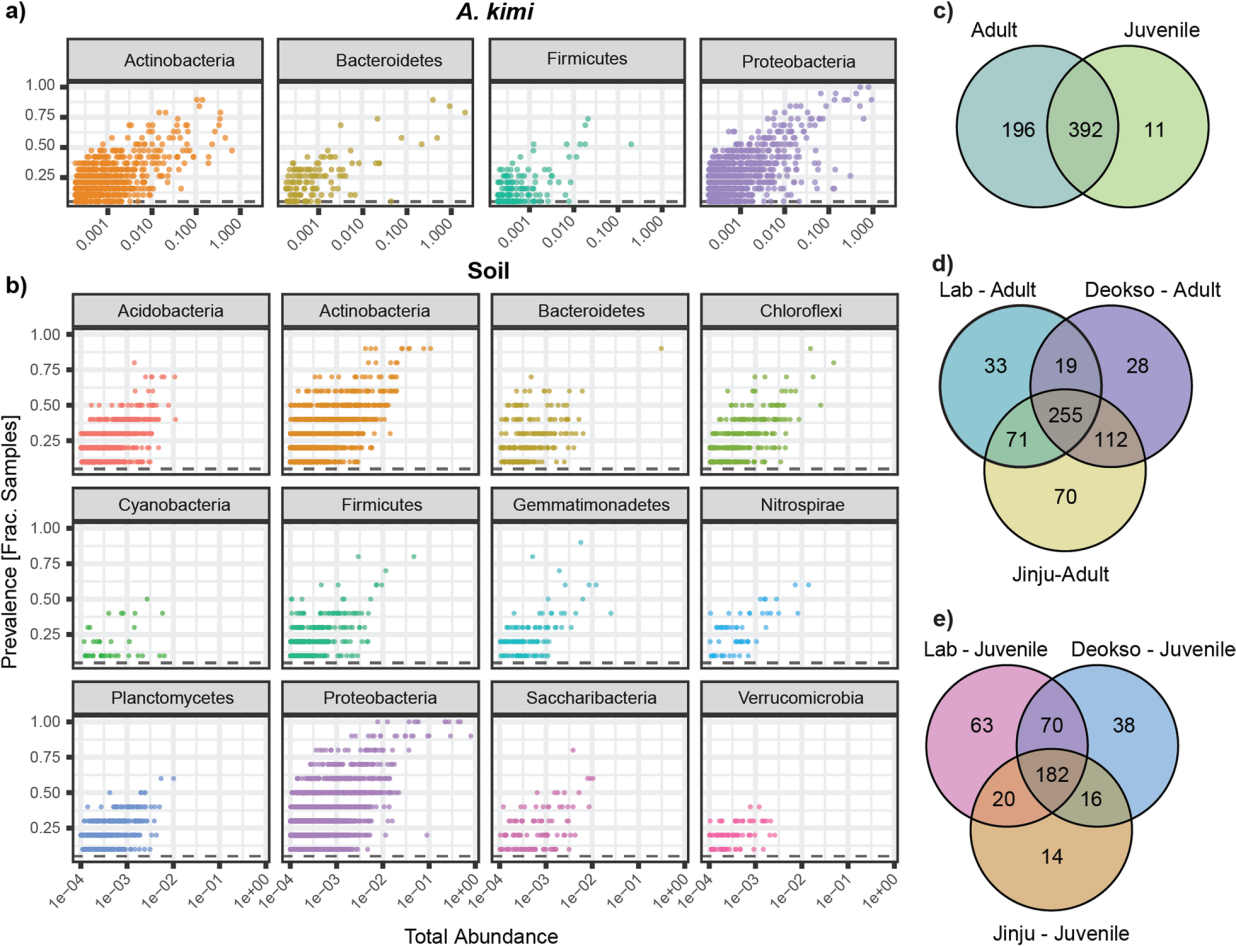
Taxonomic composition of the *A. kimi* and soil microbiota. Taxa prevalence vs total abundance graph for bacterial phyla detected in **a** *A. kimi* and **b** soil after filtering the low abundant taxa. Bacterial OTUs with > 0.01% relative abundance that were unique and shared between **a** adult and juvenile *A. kimi* samples, **b** among lab-grown and soil-grown adult *A. kimi* samples, and **c** among lab-grown and soil-grown juvenile *A. kimi* samples

We sequenced 13 adult *A. kimi* samples (3 culture conditions × 4 sampling times, and adults at day zero) and 10 soil samples (2 culture conditions × 5 sampling times) including day zero. Juveniles were found only in sampling time points at 4 and 8 weeks (Fig. 1b) and 6 juvenile *A. kimi* samples (3 culture conditions × 2 sampling times) were sequenced. Adult and juvenile individuals were distinguished based on their morphological characteristics (Additional file 1: Fig. S1). We obtained a total of 2,712,257 reads for *A. kimi* with an average of 142,750 (± 92,271) (mean ± SD) reads per sample, and 1,407,805 reads for soil, with an average of 140,780 (± 64,857) (mean ± SD) reads per sample. The mock community generated 58,091 reads, and we identified all the bacterial strains in the mock community at the expected proportions. The prevalence of key phyla in *A. kimi* adults and juveniles and soil samples provided the first insight into the abundant taxa in our data set (Fig. 2a, b). Proteobacteria, Actinobacteria and Bacteroidetes, and Firmicutes were the dominant bacteria phyla common to both *A. kimi* and soil. Additional bacterial phyla, including Acidobacteria, Saccharibacteria, Chloroflexi, and Planctomycetes, were also detected in soil samples at relatively high abundance. We analyzed the shared and unique OTUs with > 0.01% relative abundance within and between the different groups of samples (Fig. 2c–e). The majority of the OTUs (392 OTUs) found in *A. kimi* were shared not only between adults and juveniles, but also across the culture conditions: laboratory-grown and two soil environments (Deokso and Jinju).

We estimated the alpha diversity of *A. kimi* and soil samples using the observed OTUs, Chao1, Shannon, and Inverse Simpson indices (Additional file 1: Fig. S2). Alpha diversity matrices were not significantly different among *A. kimi* samples. The alpha diversity of *A. kimi*, either belonging to different developmental stages or grown in different culture environments, showed no differences (*p* > 0.1) (Additional file 1: Figs. S3 and S4). However, the alpha diversity matrices were varied between soil environments. The observed OTU and Chao1 indices of the Deokso soil was significantly higher than that of the Jinju soil (*p* < 0.01) while the Shannon and Inverse Simpson indices showed no significant difference (Additional file 1: Fig. S5). The alpha diversity of the soil in terms of Shannon and Inverse Simpson indices was significantly higher than that of *A. kimi* (*p* < 0.001) but observed OTU and Chao1 indices showed no significant difference (Additional file 1: Fig. S6).

### Comparison of bacterial community of adult and juvenile *A. kimi*: a shift during the growth

**Fig. 3:**
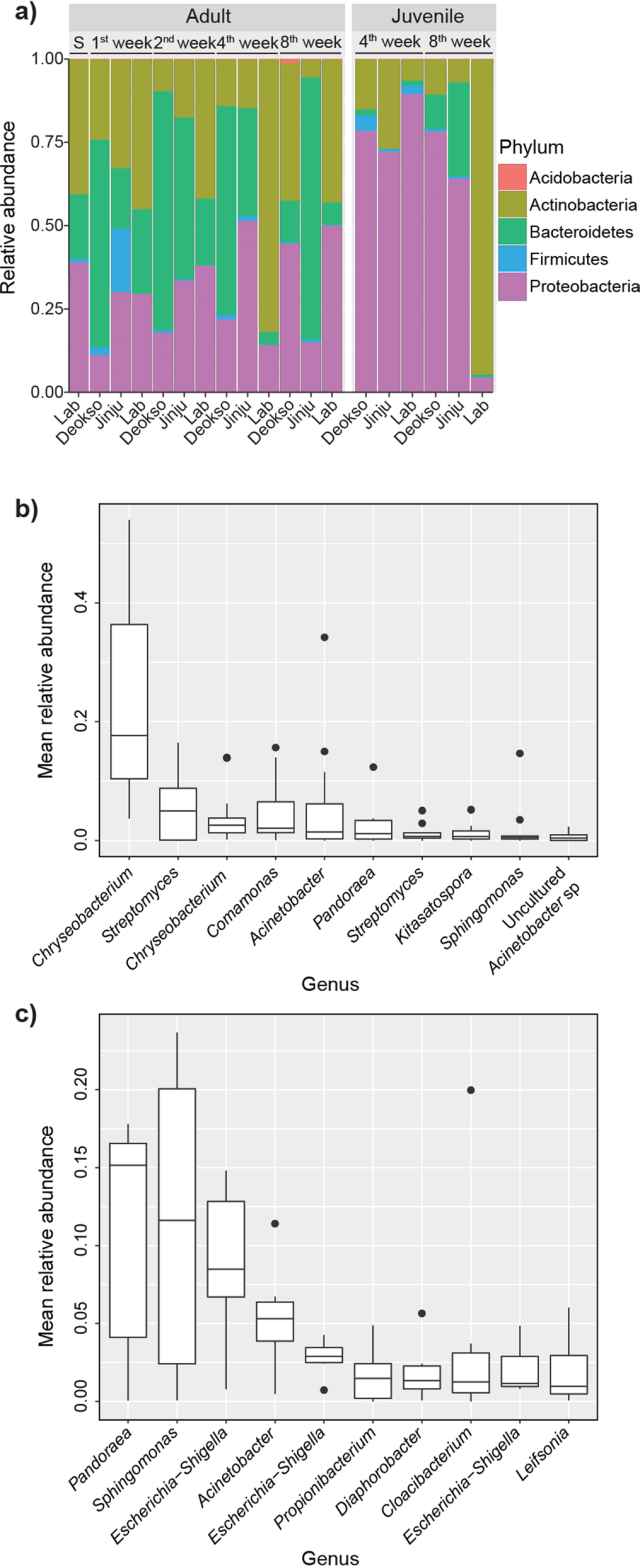
Microbial community composition in adult and juvenile *A. kimi*. **a** Relative abundance of different phyla (relative abundance > 0.001%) in adult and juvenile *A. kimi.* Relative abundance of the 10 most abundant OTUs resolved at the genus level in, **b** adult *A. kimi* and **c** juvenile *A. kimi*

While the dominant phyla in *A. kimi* were identified as Actinobacteria, Proteobacteria and Bacteroidetes representing > 97% of the whole *A. kimi* microbiota (Fig. 3a), the relative proportion of these three phyla was varied between the microbiota of juvenile and adult *A. kimi*. The relative proportions of the three abundant phyla were mostly consistent in the adult microbiota in various sampling conditions, revealing Bacteroidetes as the dominant phylum in general. The microbiota of juveniles showed a similar community composition in all samples (lab, Deokso and Jinju) at the time of first sampling of the juveniles in the fourth week. However, at the second sampling in the eighth week, the dominant phylum was changed in lab-grown juveniles. Proteobacteria was dominant in all soil-grown juveniles whereas Actinobacteria was most abundant in lab-grown juveniles in the eighth week sample (Fig. 3a). When the taxonomic ranks of the dominant OTUs were resolved at the genus level, *Chryseobacterium, Streptomyces, Comamonas,* and *Acinetobacter* were appeared as the most abundant genera in the microbiota of adult *A. kimi* (Fig. 3b). Similarly, *Pandoraea, Sphingomonas, Escherichia–Shigella*, and *Acinetobacter* were identified as the most abundant genera in juvenile *A. kimi* (Fig. 3c).

**Fig. 4:**
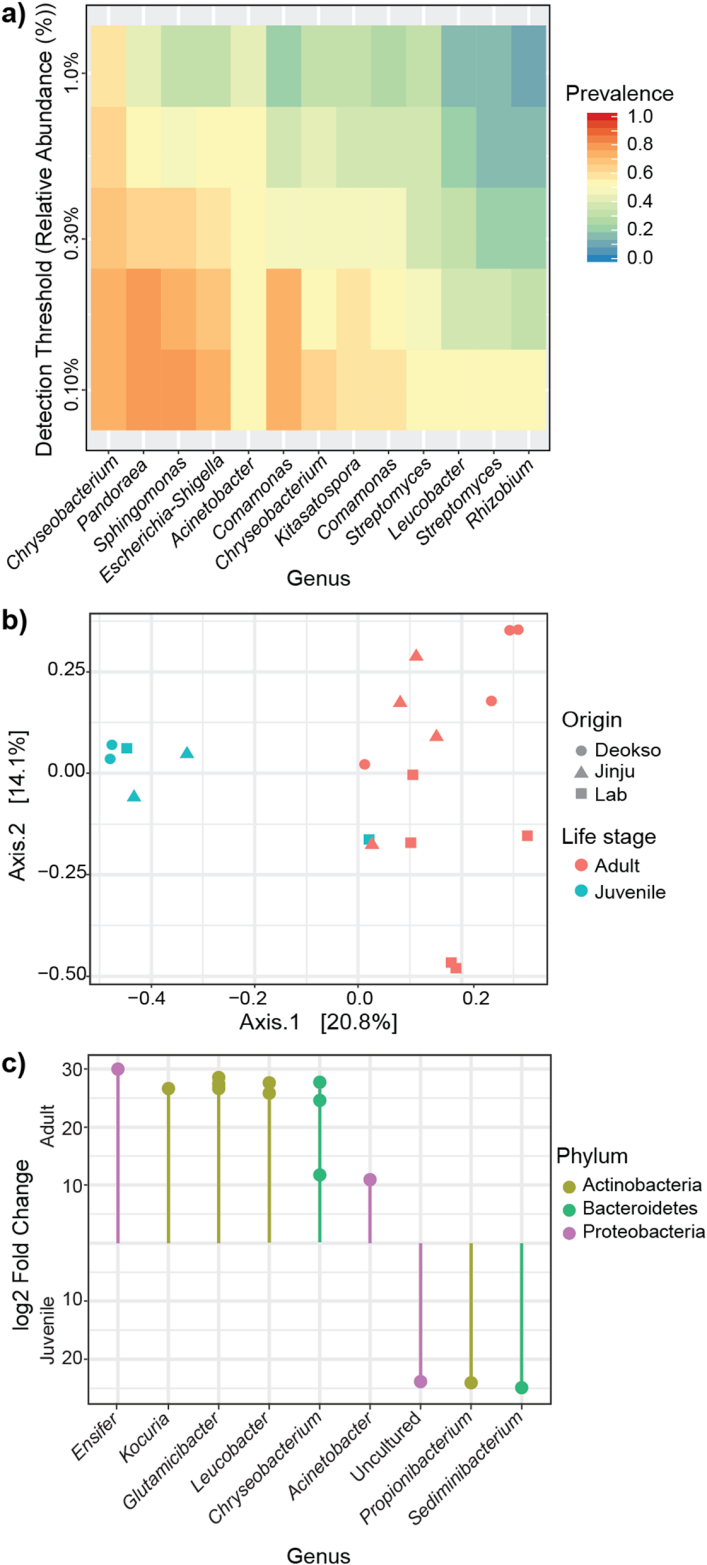
The core microbiota and comparison of the adult and juvenile *A. kimi* microbiota. **a** The core microbiota of adult and juvenile *A. kimi* with over 0.1% relative abundance and over 50% prevalence thresholds consisted of 13 OTUs. **b** Ordination analysis to visualize the differences between adult and juvenile microbiota. The multidimensional scaling plot was based on the Bray–Curtis dissimilarity matrix. **c** Differentially abundant bacterial taxa in adult and juvenile samples. Differentially abundant genera were shown as the log2 fold change. Bacterial taxa showing significantly high abundance (*p* < 0.001) in adult or juvenile *A. kimi* are indicated

The core microbiota, which is stable and abundant members of the bacterial community [5, 16, 56], of adult and juvenile *A. kimi* consisted of 13 OTUs belonging to *Chryseobacterium*, *Pandoraea, Sphingomonas, Escherichia–Shigella,* and *Acinetobacter* (at 0.1% relative abundance and 50% prevalence threshold) (Fig. 4a). Ordination analysis of adults and juveniles, using the Bray*–*Curtis dissimilarity distance, visualized that the adult and juvenile groups clustered separately except that lab-grown 4-week-old juveniles that came into a cluster with lab-grown adult samples (Fig. 4b). The changes in the bacterial composition of adults and juveniles were tested using the Permutational Multivariate Analysis of Variance (PERMANOVA) on the Bray*–*Curtis dissimilarity matrix. The proportions of three dominant OTUs, belonging to *Sediminibacterium* and *Propionibacterium,* were differentially increased in juvenile samples compared to adults (*p* < 0.001, R^2^ = 0.155) (Fig. 4c). Similarly, we observed that 11 OTUs belonging to 6 genera (*Chryseobacterium, Acinetobacter, Glutamicibacter, Leucobacter Ensifer,* and *Kocuria*) were differentially abundant (*p* < 0.001) in adults compared to the juveniles.

We identified 283 OTUs (with > 0.01% relative abundance) which were shared between the microbiota of adult and juvenile *A. kimi*. In addition, 95 OTUs were unique to adults, and 52 were unique to juveniles, suggesting the existence of specific microbiomes at different developmental stages (Additional file 1: Fig. S7a). OTUs belonging to Acidobacteria, Chloroflexi, Saccharibacteria and Verrucomicrobia were found exclusively in adult *A. kimi*. In contrast, Fusobacteria were only found in juveniles (Additional file 1: Fig. S7b). OTUs belonging to the orders Acidimicrobiales, Catenullisporales, Frankiales, Gaiellales, Solirubrobacterales and Streptosporangiales of the phylum Actinobacteria; Desulfurellales, Rhodospirillales and Myxococcales of the phylum Proteobacteria; and Cytophagales of the phylum Bacteroidetes were found only in adult *A. kimi.* Actinomycetales of the phylum Actinobacteria was found exclusively in juvenile *A. kimi* (Additional file 1: Fig. S7c).

### Community shift in the adult *A. kimi* microbiota under laboratory and different soil environments

**Fig. 5:**
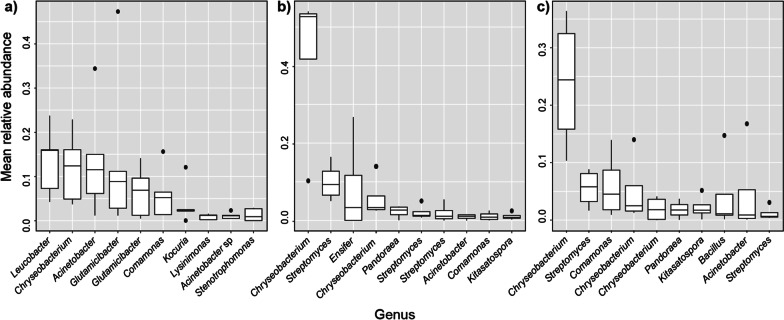
Microbial community composition in adult *A. kimi* grown under different environmental conditions. Relative abundance of the 10 most abundant bacterial OTUs resolved at the genus level in, **a** lab grown adult *A. kimi*, **b** adult *A. kimi* grown in Deokso soil and **c** adult *A. kimi* grown in Jinju soil

The dominant bacterial taxa in the microbiota of lab-grown adult *A. kimi* were different from that of the soil-grown adults*.* At the genus level, *Leucobacter, Chryseobacterium*, *Acinetobacter*, *Glutamicibacter*, and *Comamonas* were among the 10 most abundant taxa in the microbiota of lab-grown adult *A. kimi* (Fig. 5a). *Chryseobacterium, Streptomyces*, *Ensifer*, *Acinetobacter*, and *Kitasatospora* were detected in high abundance in the microbiota of adult *A. kimi* grown in Deokso soil (Fig. 5b). *Chryseobacterium, Streptomyces, Comamonas, Kitasatospora,* and *Sphingomonas* were among the most abundant taxa in adult *A. kimi* grown in Jinju soil (Fig. 5c).

**Fig. 6:**
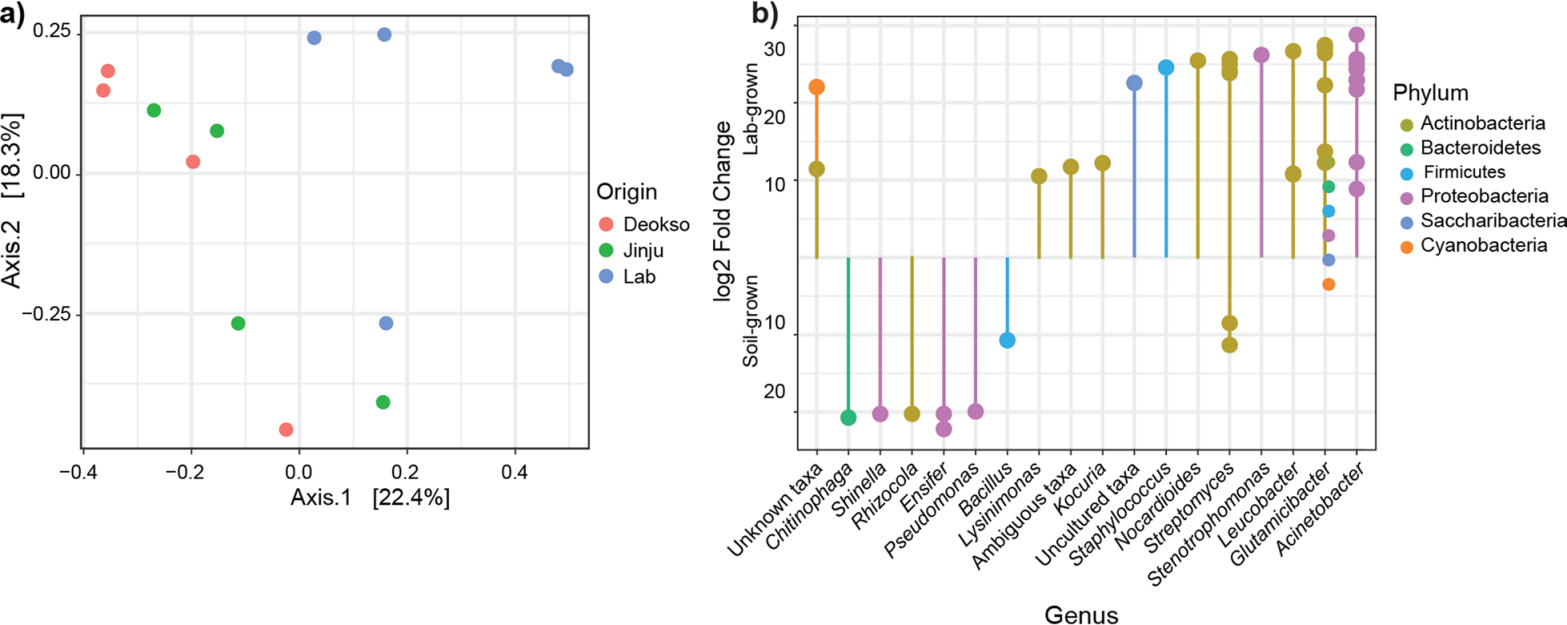
The comparison of adult *A. kimi* grown under different environmental conditions. **a** Ordination analysis to visualize the difference between lab-grown and soil-grown adult *A. kimi*. The multidiamentioanl scaling plot was based on the Bray–Curtis dissimilarity matrix. **b** Differentially abundant bacterial taxa in lab-grown and soil-grown adult *A. kimi* groups. Differentially abundant genera were shown as the log2 fold change. Bacterial taxa showing significantly high abundance (*p* < 0.001) in soil- or lab-grown *A. kimi* are indicated

Ordination analysis using the Bray–Curtis dissimilarity distances indicated that soil-grown adult *A. kimi* clustered separately from lab-grown adults (Fig. 6a). A shift in the bacterial community composition between soil- and lab-grown samples was observed in the PERMANOVA based on the Bray–Curtis dissimilarity matrix (*p* < 0.01, R^2^ = 0.183). The changes in abundant taxa in soil- and lab-grown adult *A. kimi* were identified. We observed 9 OTUs, including *Ensifer*, *Streptomyces*, *Pseudomonas*, and *Bacillus*, that showed significantly high differential abundance (*p* < 0.001) when *A. kimi* were grown in soil. Similarly, 32 OTUs have significantly high differential abundance (*p* < 0.001) in lab-grown *A. kimi*, including *Acinetobacter*, *Glutamicibacter*, and *Leucobacter* and *Nocardioides* (Fig. 6b)*.*

To evaluate the effect of soil environments on *A. kimi*, the bacterial community in the soil environments were compared to the microbiota of *A. kimi* grown in the given soils. Proteobacteria was the dominant phyla in both Deokso and Jinju soils, followed by Actinobacteria and Bacteroidetes (Additional file 1: Fig. S8a). The core microbiota of two soil environments (Deokso and Jinju) consisted of 20 OTUs (with 0.1% relative abundance and 50% prevalence thresholds) (Additional file 1: Fig. S8b). The statistically significant difference in the community composition was not found between the two groups of soil samples in the PERMANOVA based on the Bray–Curtis dissimilarity matrix (Additional file 1: Fig. S8c).

We investigated the bacterial taxa that were shared between the given soil environment and soil-grown *A. kimi* but not shared with lab-grown *A. kimi*. The presence of such taxa might suggest bacterial transfers from the soil to the *A. kimi* microbiota. Bacterial taxa belonging to the Acidobacteria, Chloroflexi, Planctomycetes, and Verrucomicrobia were exclusively found in soil-grown adult *A. kimi* (Additional file 1: Fig. S9a). OTUs belonging to the orders Acidimicrobiales, Micromonosporales, Streptosporangiales, and Solirubrobacterales of the phylum Actinobacteria; Caulobacterales and Myxococcales of the phylum Proteobacteria; and Cytophagales of the phylum Bacteroidetes were also found in soil-grown adult *A. kimi* (Additional file 1: Fig. S9b). We identified 118 OTUs (> 0.01% relative abundance) that were likely transferred from Deokso soil to Deokso soil-grown adult *A. kimi* microbiota. *Ensifer* was the most abundant genus having 6.7% mean relative abundance in *A. kimi* microbiota, followed by 11 OTUs, including *Bacillus, Streptomyces, Lentzea* and *Mycobacterium*, having > 0.1% mean relative abundance*.* Similar to Deokso soil-grown samples, 122 OTUs (> 0.01% relative abundance) were identified to be transferred from Jinju soil to Jinju soil-grown adult *A. kimi.* The 12 OTUs (> 0.1% mean relative abundance) belonged to *Bacillus* (3.7% relative abundance), *Enterobacter, Acinetobacter, Ensifer* and *Mycobacterium.* The absence of these soil-originated bacterial OTUs in the microbiota of lab-grown *A. kimi* implies that these bacteria were transferred to the *A. kimi* after they were exposed to the soil environment.

### Microbial co-occurrence network analysis in the microbiota of adult *A. kimi*

**Fig. 7:**
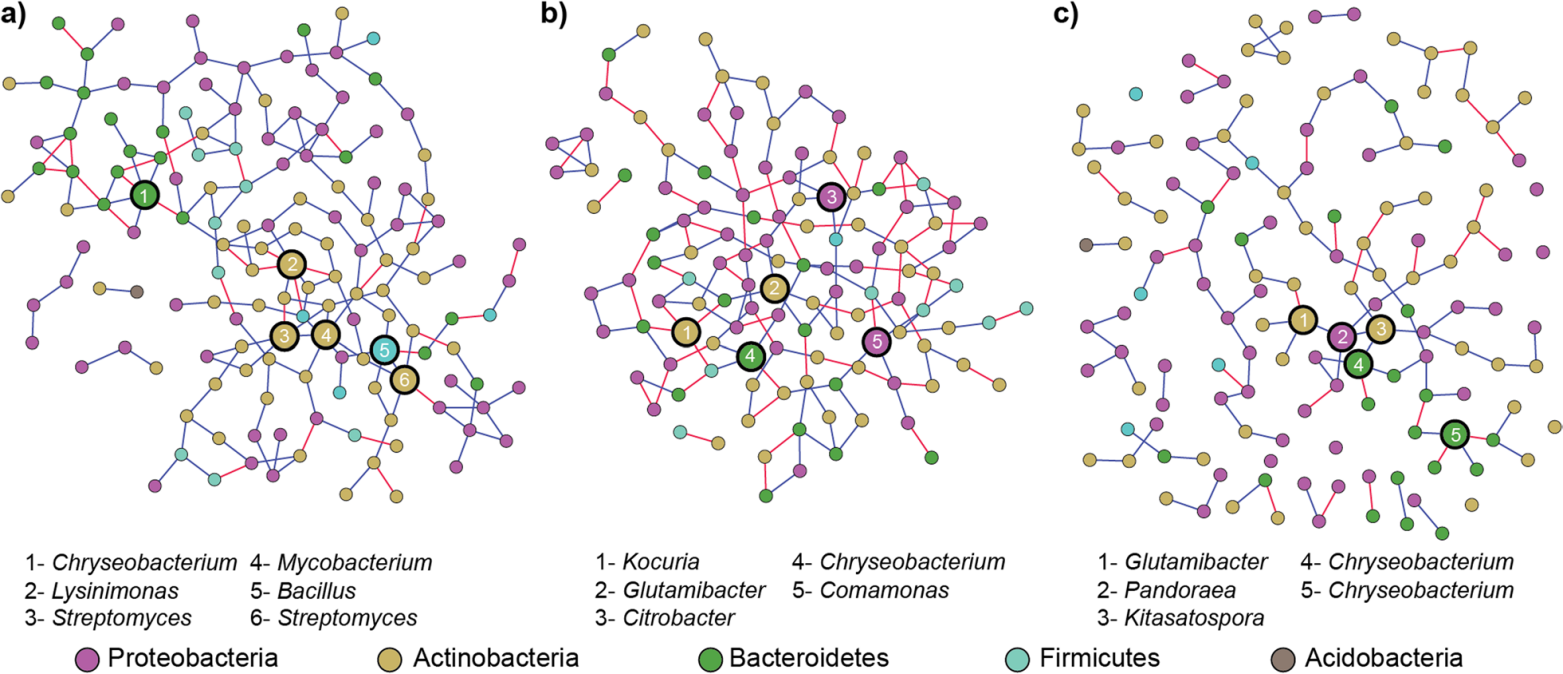
Network analysis of the bacterial taxa in the adult *A. kimi* microbiome. Bacterial network in the adult *A. kimi* microbiome was constructed using the SParse InversE Covariance estimation for Ecological Association Inference (SPIEC-EASI). **a** The overall bacterial co-occurrence network in lab-grown, and soil-grown adult *A. kimi*. **b** Bacterial co-occurrence network in lab-grown adult *A. kimi*, **c** bacterial co-occurrence network in soil-grown adult *A. kimi*. Nodes representing corresponding OTUs and two OTUs with significant co-association were linked by an edge. Node color represents the phylum to which the OTU belongs. Positive associations are colored with a blue edge and negative associations with red. Large circles indicate the putative keystone taxa in each network. OTUs with the highest degree, high closeness centrality, and low betweenness centrality were selected as putative keystone taxa

To search the patterns of co-occurrence of bacterial taxa in the adult *A. kimi* microbiota, we employed the SParse InversE Covariance estimation for Ecological Association Inference (SPIEC-EASI) analysis [30]. The overall bacterial co-occurrence network in adult *A. kimi* (lab-grown and soil-grown combined) consisted of 146 nodes (with > 0.01% relative abundance) and 182 edges (Fig. 7a). The overall network structure showed more positive than negative co-associations. To examine the structure and connectivity of the microbial network, we calculated the network density (defined as the ratio of realized to possible number of edges), the clustering coefficient (defined as the probability that nodes close to a given node are connected), and the average number of neighbors (defined as the average number of edges per node) [28]. The co-occurrence network for adult *A. kimi* microbiota has a network density of 0.017, a clustering coefficient of 0.089 and 2.493 neighbors on average. We selected six OTUs with the highest degree, high closeness centrality and low betweenness centrality in the overall network as putative keystone taxa. These putative keystone taxa identified in the co-occurrence network included both high abundance OTUs (*Chryseobacterium* and *Streptomyces*) and low abundance OTUs (*Mycobacterium* and *Lysinimonas*). This observation suggested the importance of low abundance taxa to the structure of the microbial network.

Since the bacterial community compositions of lab-grown and soil-grown adult *A. kimi* were different, we built the bacterial co-occurrence networks using lab-grown and soil-grown adult *A. kimi* microbiota independently. The co-occurrence network of lab-grown adult *A. kimi* microbiota consisted of 120 nodes and 152 edges (Fig. 7b), whereas that of soil-grown adult *A. kimi* had 137 nodes and 114 edges (Fig. 7c)*.* The average number of neighbors was 2.5 for the network of lab-grown adult *A. kimi* microbiota, and 1.7 for that of the soil-grown. The clustering coefficient of the network of lab-grown adult *A. kimi* microbiota was 0.043 while that of soil-grown adult *A. kimi* microbiota was 0.005. We also observed that the network density of lab-grown adult *A. kimi* microbiota (0.021) was higher than that of soil-grown (0.012)*. Chryseobacterium* and *Glutamicibacter* were common keystone taxa in both networks. The degree of the keystone taxa in soil-grown adult *A. kimi* microbiota was lower than that of lab-grown, indicating that the established microbial network in lab-grown adult *A. kimi* was perturbed by the soil bacteria that colonized in *A. kimi* and altered the previously established bacterial network structure.

## Discussion

*Allonychiurus kimi* (Lee) is widely being used in the Republic of Korea as a test species in ecotoxicological studies. Its adaptation to the physicochemical properties of Korean soil makes *A. kimi* a better candidate in the Republic of Korea than *F. candida*, which is widely being used worldwide as a test organism. Although several studies have been conducted into the microbiota of *F. candida* and other Collembola species, including *Orchesella cincta* and *Orchesellides sinensis* [1, 2, 35], the composition of the microbiota of *A. kimi* is still poorly understood. Here, we focused on the microbiota of a laboratory-maintained *A. kimi* population, and the way in which the microbiota changes across developmental stages and when exposed to new environmental conditions. The bacterial community composition of *A. kimi* is similar to that of other Collembola species reported in the literature [1, 2, 35]. The microbiota of laboratory-maintained *F. candida* consists of four main phyla: Actinobacteria, Bacteroidetes, Proteobacteria, and Firmicutes [57], which is similar to the microbiota of *A. kimi* but relative abundance of Firmicutes was lower than that of *F. candida*, *O. cincta* or *O. sinensis*. These four main phyla are also known as the core microbiota of other soil-dwelling animals, such as earthworms and nematodes [9, 38]. The genus *Chryseobacterium* found as a dominant genus in *A. kimi* was previously reported in another Collembola species, *Orchesella cincta,* at high abundance across body sites but was not found in *F. candida* [6].

Wolbachia is known as a common symbiont in certain Collembola species [1]. It is noteworthy that, in our study, the microbiota of the laboratory-maintained *A. kimi* has very low abundance of *Wolbachia.* Since we did not compare the microbiota of wild *A. kimi* with those of laboratory-maintained organisms, we were unable to determine whether the low abundance of *Wolbachia* is a consequence of continuous maintenance under laboratory conditions. However, it has been reported that certain other Collembola species also have a low abundance of *Wolbachia* in their microbiota [51].

Comparison of adult and juvenile *A. kimi* microbiota suggested that both hereditary and environmental factors affect the *A. kimi* microbiota. Spatial and temporal variation in the insect gut microbiome across host developmental stages has previously been reported [3, 4]. However, microbiome studies on Collembola have been mainly confined to adults. We showed that the microbiota of adult and juvenile *A. kimi* had different compositions depending on the developmental stage (Fig. 2). The juvenile microbiota was dominated by Proteobacteria, whereas Bacteroidetes were dominant in adults. We suggest that the similar bacterial community composition of *A. kimi* juveniles at the birth, irrespective of the growth environment, might be a consequence of vertical transmission of the parental *A. kimi* microbiota in establishing the juvenile microbiota. Later, environmental factors such as nutrients, humidity, temperature, chemical/physical composition of soil played key role in shaping the juvenile microbiota into its adult composition. We assume that the complex process of transforming the juvenile microbiota into adult microbiota is similar to what is described in the literature [11, 17].

The microbiota of soil dwelling animals can therefore be affected not only by inherent host related factors, but also by environmental factors [3, 16]. The effects of environmental factors on the microbiota are transient and subject to dynamic change [4]. We observed that the bacterial community composition of adult *A. kimi* gradually changed when they were grown in soil. Actinobacteria were abundant in lab-grown adult *A. kimi,* whereas Bacteroidetes dominated in soil-grown adults. These observations suggest that the complex environmental factors play a pivotal role in altering the host microbiota. The members of the soil microbiota may alter the host microbiota by colonizing the host. In addition, different dietary patterns in the two groups can also affect the microbiota, since the diet is a key factor in altering the Collembola microbiota [53]. These were supported by previous studies which emphasized the effects of environmental factors in altering the host-associated microbiota in Collembola [27, 54].

The *A. kimi* test species, that we used in this study, was reared under consistent laboratory conditions (diet, humidity, temperature etc.) for 20 years. Therefore, it can be speculated that the native microbiota, which was present in the wild *A. kimi*, has changed throughout this period, and the laboratory-maintained species has a well-established microbial network. And this microbial network could be different from that of the native soil-dwelling wild species. We hypothesized that the established microbial network of laboratory-maintained *A. kimi* could be perturbed once the organisms were introduced into the soil. Analysis of the microbial networks can provide insights about how bacterial community structure changes in response to environmental factors [19]. Several bacterial taxa were found exclusively in soil-grown *A. kimi,* suggesting that these bacteria were transferred from the soil to *A. kimi* and colonized. This colonization may perturb the existing microbial network in *A. kimi* for a certain period until it re-stabilizes. Distortion of the existing microbial network of lab-grown *A. kimi* was explained by the network parameters such as network density, clustering coefficient and average number of neighbors. All three parameters obtained for the microbial network in soil grown *A. kimi* were lower than that of lab-grown *A. kimi*. This evidence suggested that soil microbiota drive structural changes in the established microbial network of lab-grown *A. kimi.*

The keystone taxa are defined as taxa that are highly connected and contribute individually or in association with other taxa to the structure and function of the microbiome irrespective of their abundance [7]. We identified the keystone taxa such as *Chryseobacterium* and *Glutamicibacter* in both lab-grown and soil-grown *A. kimi* microbial networks (Fig. 6)*. Chryseobacterium* was recognized as a potential pathogen in insects, including bark beetles [25]. *Glutamicibacter* was suggested to aid host metabolism [34]. Although *Kitasatospora* and *Pandoraea* were not found in high abundance, we identified them as putative keystone taxa in the microbial network of the soil-grown *A. kimi.* This finding suggested that taxa of low abundance may be significant in shaping the network structure of the microbiota. However, empirical evidence emphasizing the importance of these keystone taxa in Collembola were hardly found in the literature and further study is required to validate the importance of the low abundance keystone taxa.

The lack of microbiome data for *A. kimi* has been a long-lasting gap in the assessment of the role of the *A. kimi* microbiota in ecotoxicological studies. Since the bacterial community composition was characterized in this study, *A. kimi* can be used not only as a model organism for ecotoxicological studies, but also as a gnotobiotic-type model organism with which to study the contribution of the microbiota to its ecotoxicological effects. Our study provided useful insights into the bacterial community composition of laboratory-maintained *A. kimi* and the potential spatio-temporal changes in the microbiota under field conditions. *Allonychiurus kimi* microbiota is permissive, allowing members of the soil microbiota to colonize in *A. kimi* and form dynamic associations with the soil microbiota. Therefore, the microbiota restructuring patterns in *A. kimi,* upon exposure to different environmental conditions, can also be used as an indicator of ecotoxicological effects. Since the *A. kimi* microbiota is amenable to both laboratory and field conditions, our study will pioneer the role of the host-associated microbiota in the ecotoxicological effects exerted by this tiny soil dwelling arthropod, *A. kimi.*

## Supplementary Information

**Additional file 1**. Supplementary figures, Fig S1–S9. **Fig S1.** Graphical representation of the life cycle of A. kimi; **Fig S2.** Alpha diversity analysis; **Fig S3–S6.** Comparison of alpha diversity matrices; **Fig S7.** Shared and unique OTUs between adult and juvenile A. kimi; **Fig S8.** Microbial community composition in Deokso and Jinju soil; **Fig S9.** Shared and unique OTUs between adult A. kimi.

## Data Availability

The raw reads generated and analyzed in this study are available in the NCBI database under the BioProject accession number PRJNA752221. Raw sequence reads from soil samples are available in the NCBI Sequence Read Archive (SRA) under accession numbers SRR15352802–SRR15352811 and raw sequence reads from *A. kimi* samples are available under accession numbers SRR15355258–SRR15355276.
