# Correction to: Soil environment reshapes microbiota of laboratory-maintained Collembola during host development

**DOI:** 10.1186/s40793-022-00414-4

**Published:** 2022-04-25

**Authors:** Duleepa Pathiraja, June Wee, Kijong Cho, In-Geol Choi

**Affiliations:** 1 Department of Biotechnology, College of Life Sciences and Biotechnology, Korea University, Seoul, 02841 Korea; 2 Department of Environmental Science and Ecological Engineering, College of Life Sciences and Biotechnology, Korea University, Seoul, 02841 Korea; 3 BK21 FOUR R&E Center for Environmental Science and Ecological Engineering, Korea University, Seoul, 02841 Korea

## Correction to: Environmental Microbiome (2022) 17:16 10.1186/s40793-022-00411-7

Following publication of the original article [1], it came to the authors' attention that the article had published with the affiliations of the corresponding authors erroneously swapped. That is, Kijong Cho had affiliation 1 instead of 2, while In Geol Choi had affiliation 2 instead of 1.

The affiliations information of the corresponding authors has been corrected in the published article, and the correct information may be found in this erratum.
