# Supplementary material for: Soil environment reshapes microbiota of laboratory-maintained Collembola during host development
Source: Environ Microbiome. 2022 Apr 5;17:16. doi: 10.1186/s40793-022-00411-7 (PMC8981701; doi:10.1186/s40793-022-00411-7)
Supplement: Supplementary file 1 — Additional file 1. Supplementary figures, Fig S1–S9. Fig S1. Graphical representation of the life cycle of A. kimi; Fig S2. Alpha diversity analysis; Fig S3–S6. Comparison of alpha diversity matrices; Fig S7. Shared and unique OTUs between adult and juvenile A. kimi; Fig S8. Microbial community composition in Deokso and Jinju soil; Fig S9. Shared and unique OTUs between adult A. kimi. [file 40793_2022_411_MOESM1_ESM.docx]

**Additional file**

**Soil Environment Reshapes Microbiota of Laboratory-maintained Collembola during Host Development**

Duleepa Pathiraja^a†^, June Wee^b†^, Kijong Cho^a*^, In-Geol Choi^b*^

^a^ Department of Biotechnology, College of Life Sciences and Biotechnology, Korea University, Seoul 02841, Korea

^b^ Department of Environmental Science and Ecological Engineering, College of Life sciences and Biotechnology, Korea University, Seoul, 02841, Korea.

**^*^** Correspondence should be addressed to In-Geol Choi (igchoi@korea.ac.kr) and Kijong Cho (kjcho@korea.ac.kr).

^†^Duleepa Pathiraja and June Wee contributed equally to this work. Author order was determined by the contribution to analyzing data and writing the manuscript.


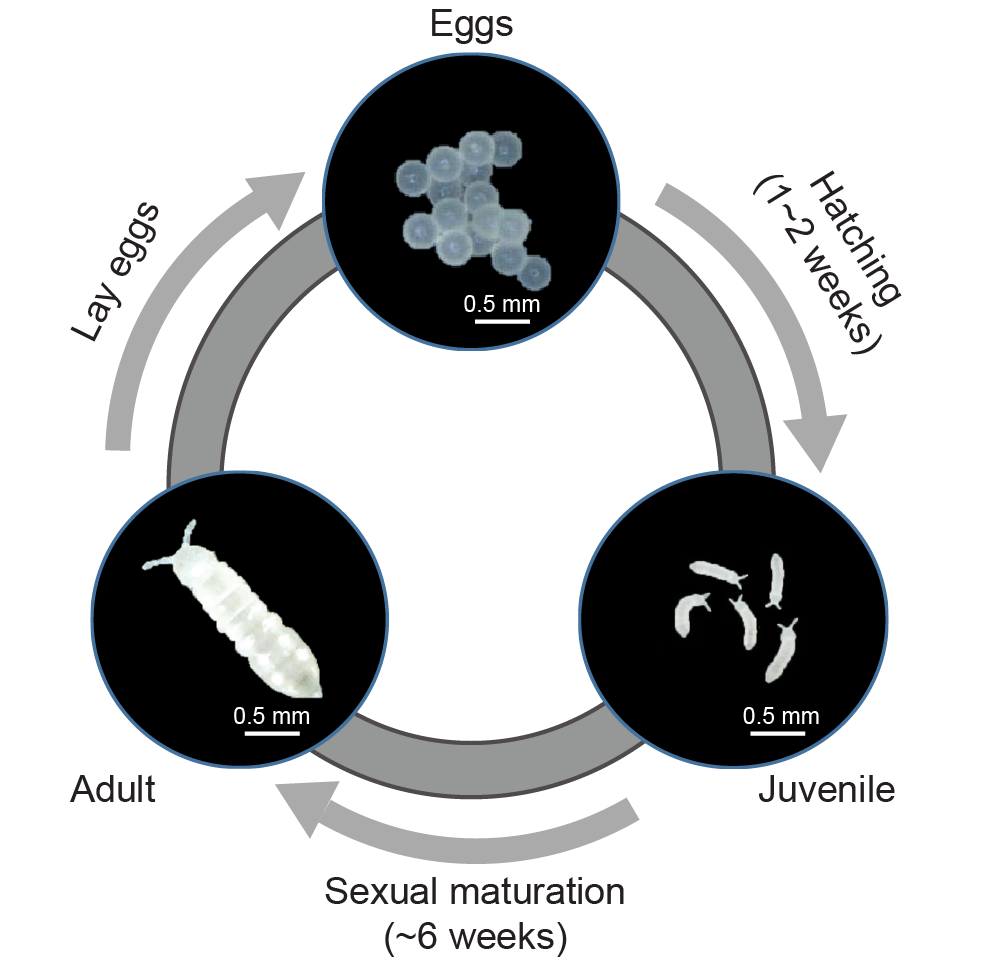


**Figure S1:** Graphical representation of the life cycle of *A. kimi.* The life cycle of *A. kimi* is divided into three main stages: eggs, juvenile, and adult. The eggs laid by adult *A. kimi* take approximately two weeks to hatch, and the juveniles become sexually mature in approximately six weeks.


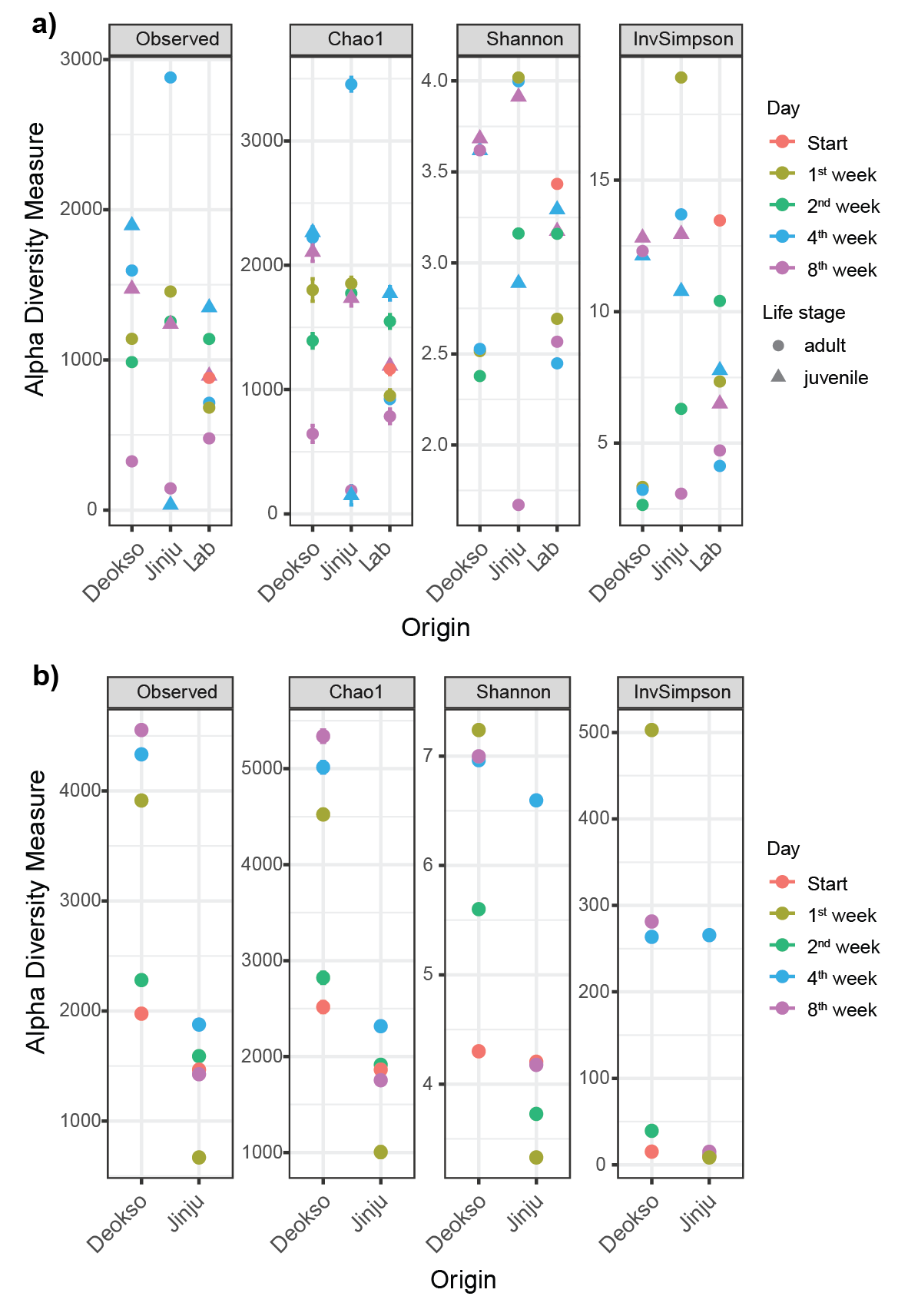


**Figure S2:** Alpha diversity analysis. Alpha diversity matrices of the a) *A. kimi* and b) soil microbiota. Observed OTUs, Chao1 index, Shannon index and Inverse Simpson index were determined for *A. kimi* and soil samples separately. Sample collection date and the life stage were indicated for *A. kimi* at three separate culturing conditions (lab, Deokso soil and Jinju soil).


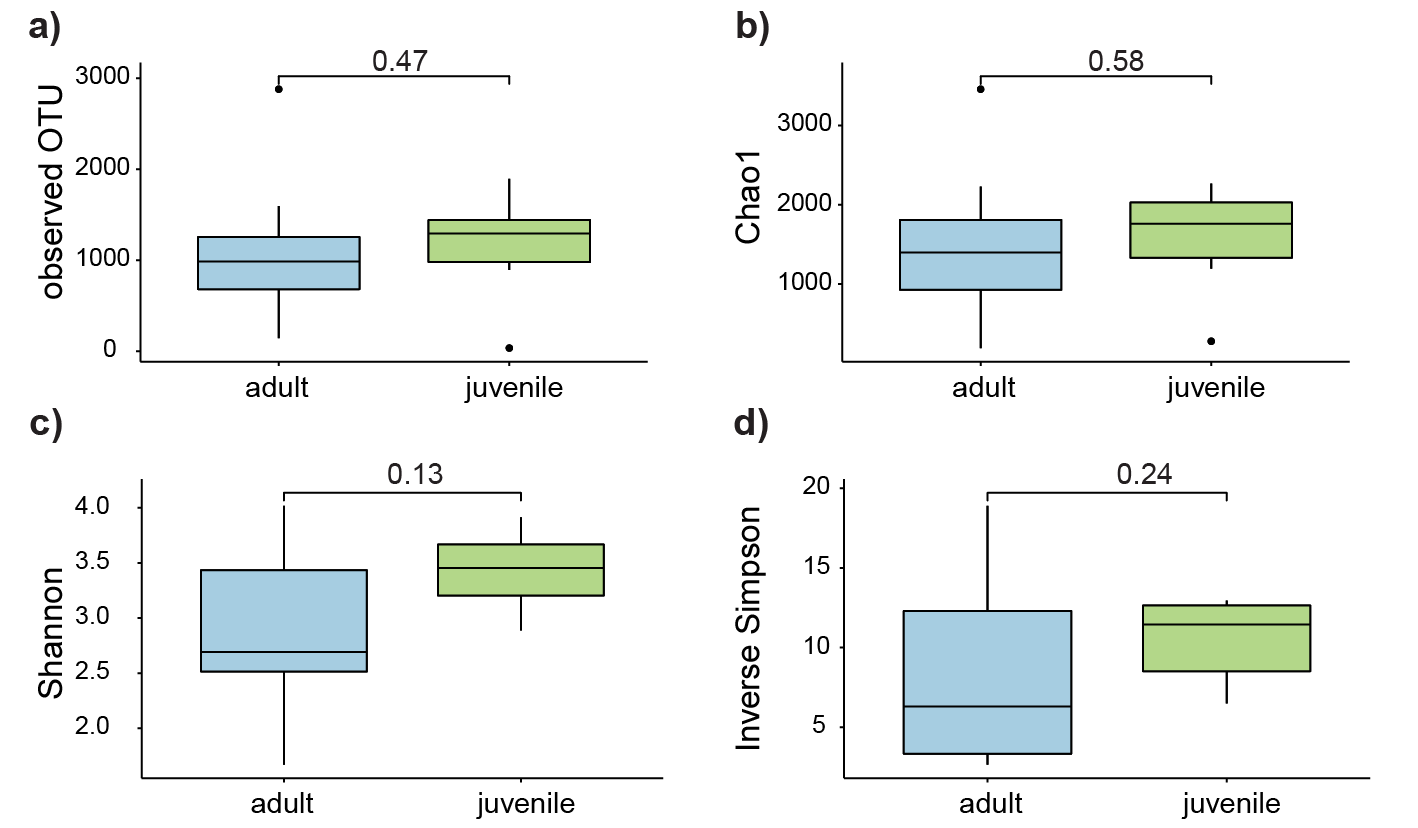


**Figure S3:** Comparison of alpha diversity matrices between adult and juvenile *A. kimi.* a) Observed OTUs, b) Chao1, c) Shannon and d) Inverse Simpson indices were used to compare the alpha diversity.


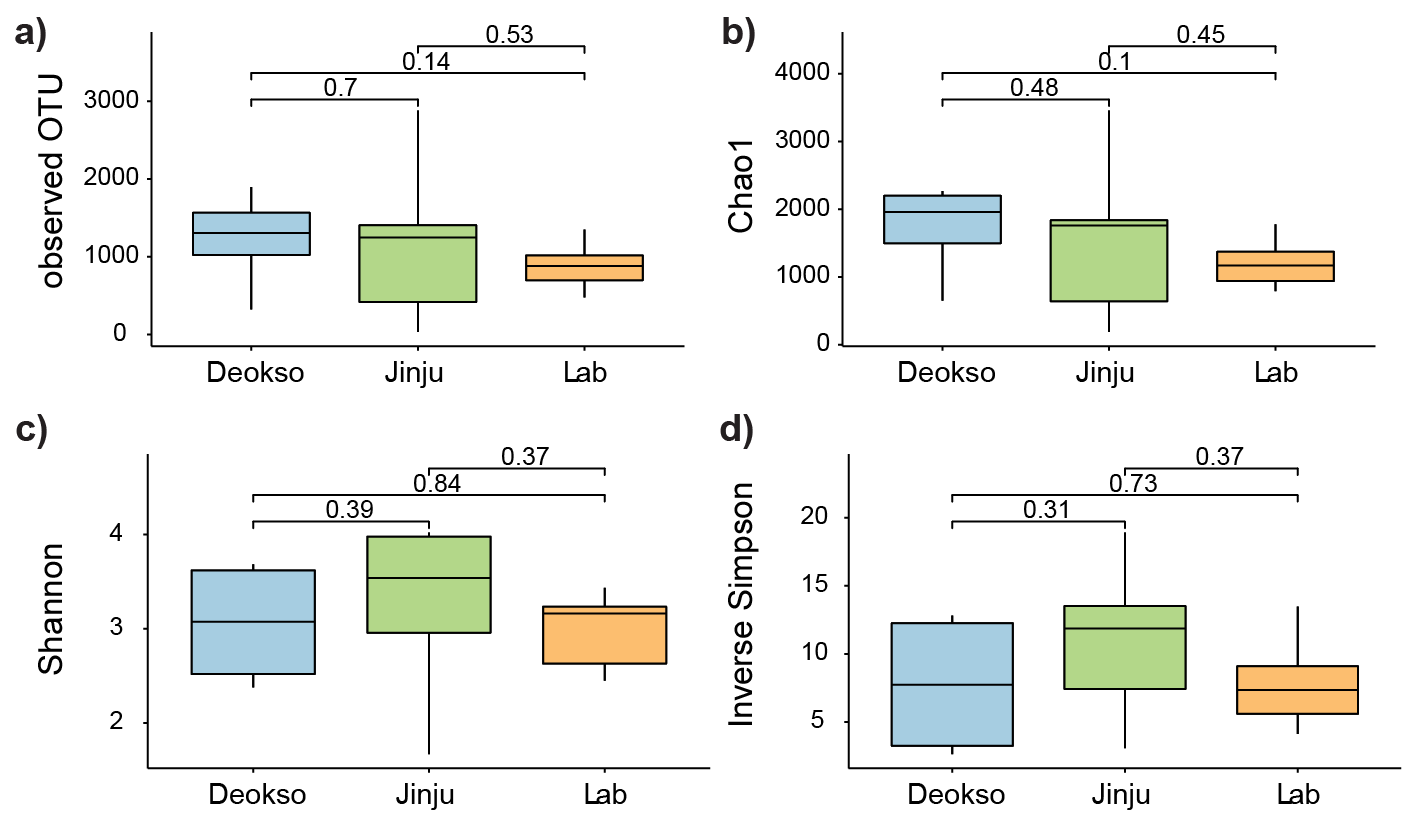


**Figure S4:** Comparison of alpha diversity matrices among adult *A. kimi* grown under different conditions (lab, Deokso soil, and Jinju soil). a) Observed OTUs, b) Chao1, c) Shannon and d) Inverse Simpson indices were used to compare the alpha diversity.


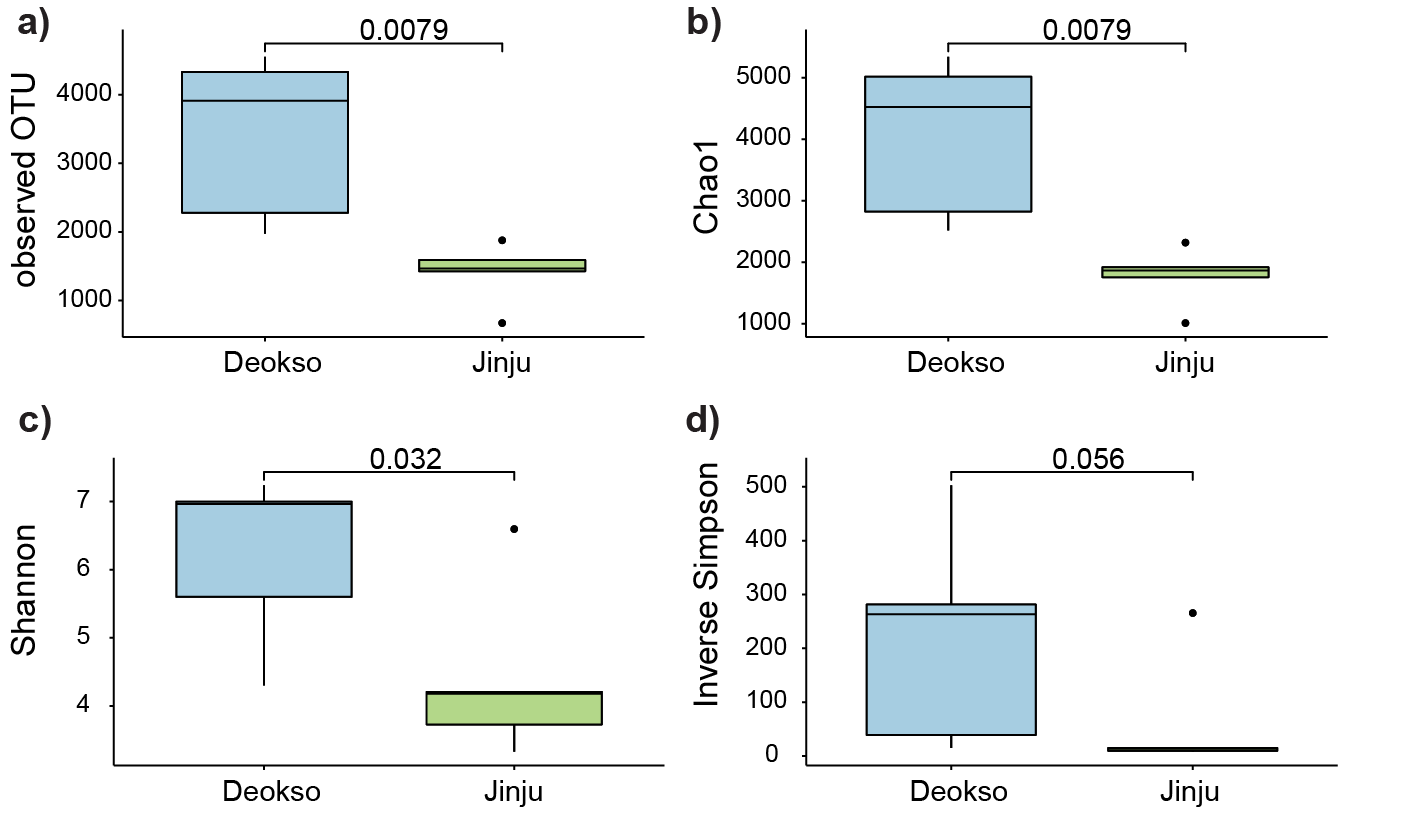


**Figure S5:** Comparison of alpha diversity matrices between Deokso and Jinju soil. a) Observed OTUs, b) Chao1, c) Shannon and d) Inverse Simpson indices were used to compare the alpha diversity. Significant difference was observed between Deokso and Jinju soil for observed OTUs and Chao1 indices (p<0.01).


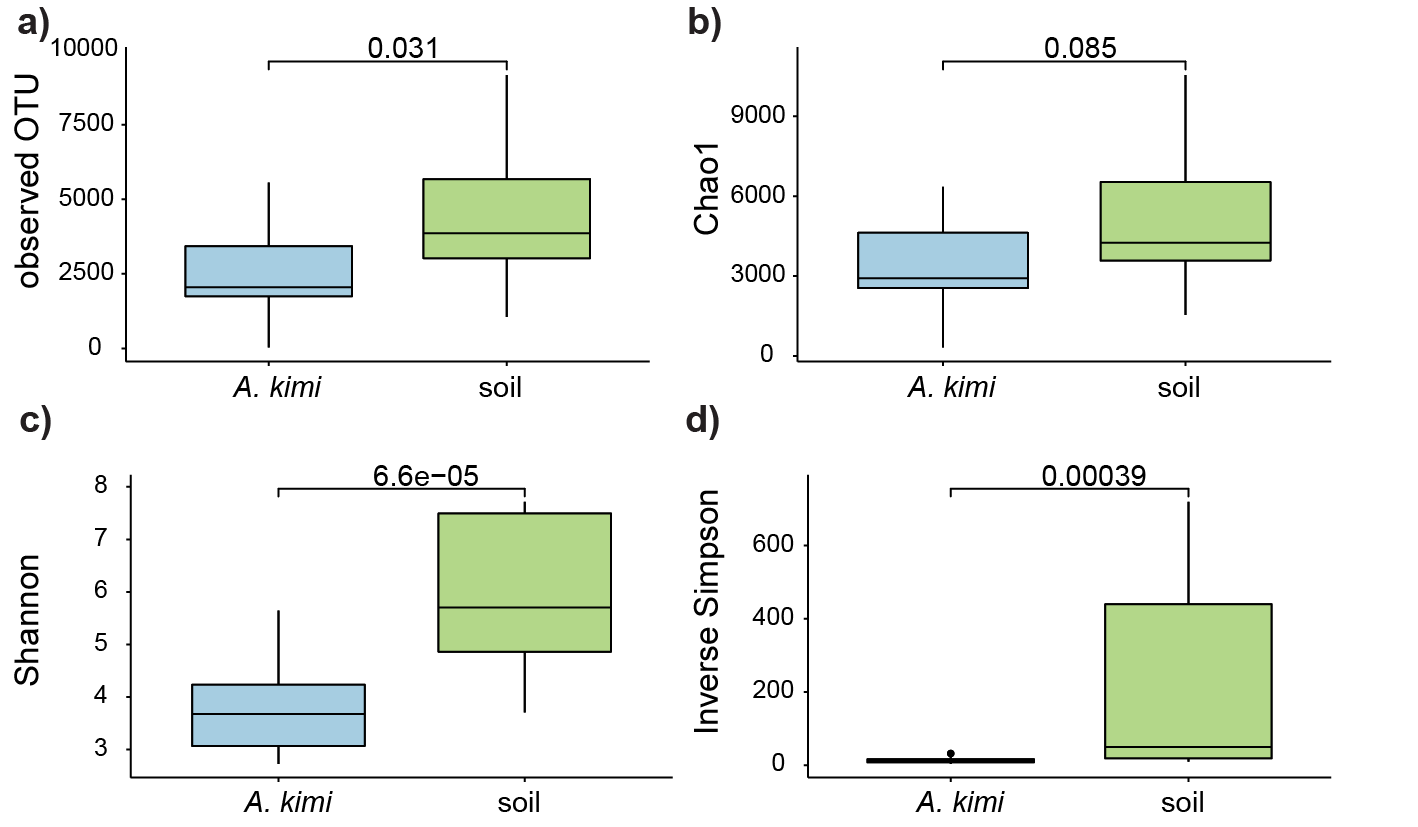


**Figure S6:** Comparison of alpha diversity matrices between *A. kimi* (adult and juvenile combined) and soil (Deokso and Jinju combined). a) Observed OTUs, b) Chao1, c) Shannon and d) Inverse Simpson indices were used to compare the alpha diversity. Significant difference was observed between *A. kimi* (adult and juvenile combined) and soil (Deokso and Jinju combined) for Shannon and Inverse Simpson indices (p<0.001).


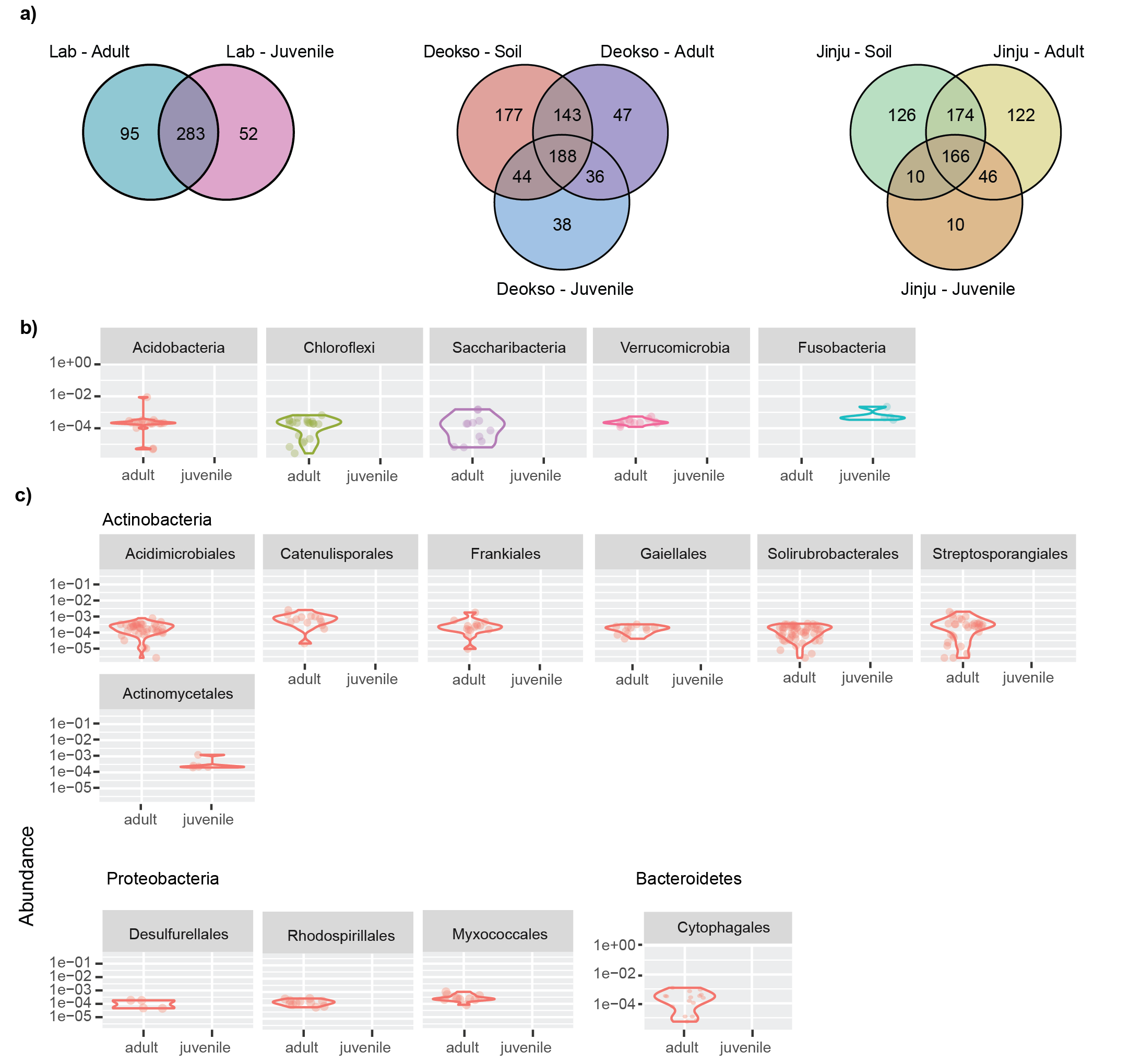


**Figure S7:** Shared and unique OTUs between adult and juvenile *A. kimi.* a) Venn diagram that represents the shared and unique OTUs between lab-grown adult and juvenile *A. kimi*, Deokso grown *A. kimi* and Deokso soil, Jinju grown *A. kimi* and Jinju soil. These Venn diagrams suggested that lab grown juvenile yeast inherited its microbiota through the adults, but both hereditary and environmental factors affect the microbiota of soil-grown juvenile *A. kimi*. b) OTUs belong to four bacterial phyla were exclusively found in adult *A. kimi* microbiota, whereas one in juvenile *A. kimi* microbiota. c) Six orders belong to phylum Actinobacteria, three orders belong to phylum Proteobacteria, and one order belong to phylum Bacteroidetes were exclusively found in adult *A. kimi.* Actinomycetales belong to phylum Actinobacteria was exclusively found in juvenile *A. kimi.*


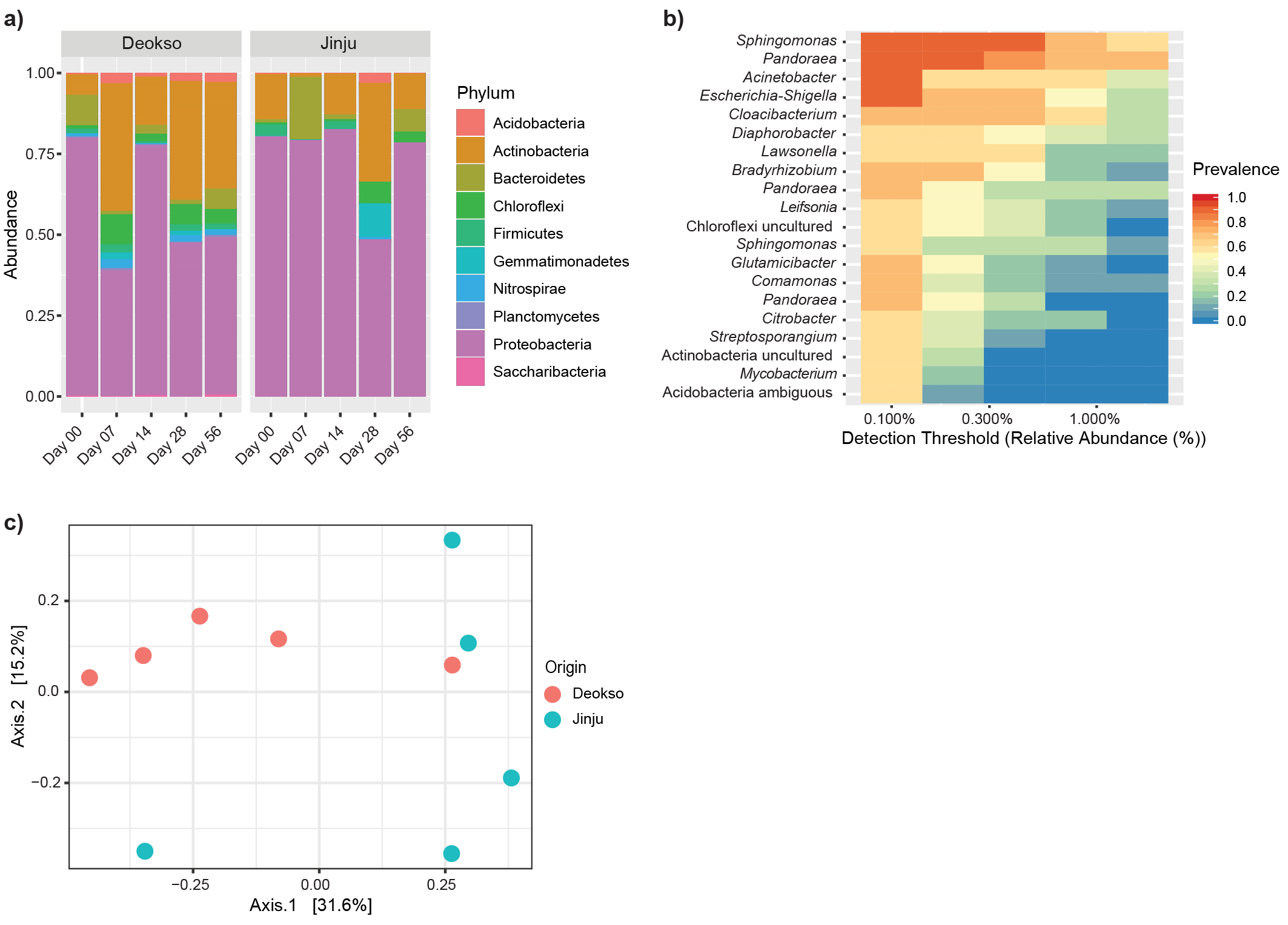


**Figure S8:** Microbial community composition in Deokso and Jinju soil*.* a) Relative abundance of different phyla (relative abundance>0.001%) in Deokso and Jinju soil*.* b) The core microbiota of soil (Deokso and Jinju) with 0.1% relative abundance and 50% prevalence thresholds consisted of 20 OTUs. c) Ordination analysis to visualize the difference between adult and juvenile microbiota. Multidiamentioanl scaling plot was based on the Bray-Curtis dissimilarity matrix. Significant difference of the microbiota was not observed between Deokso and Jinju soil.


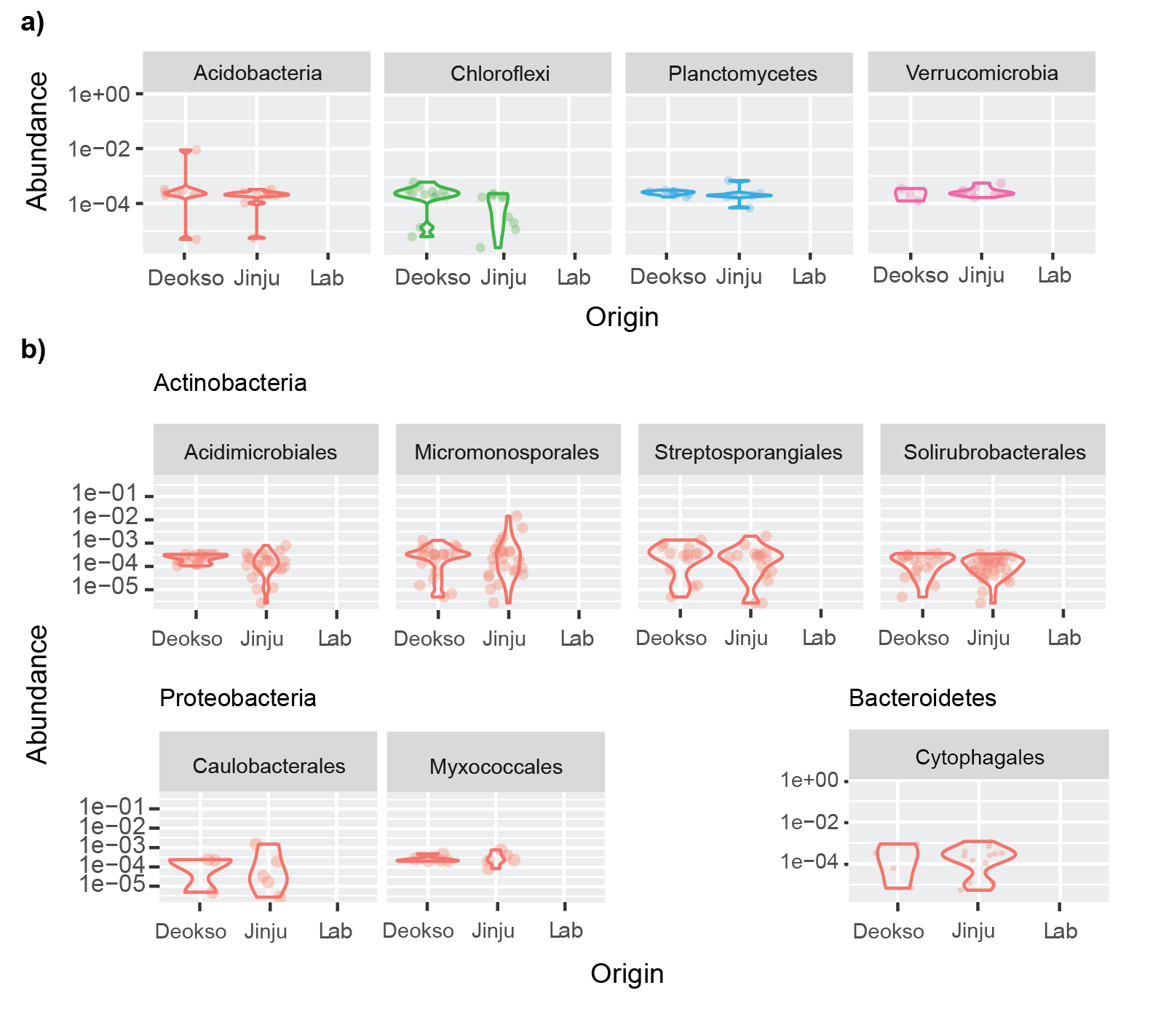


**Figure S9:** Shared and unique OTUs between adult *A. kimi.* a) OTUs belong to four bacterial phyla were exclusively found in soil grown adult *A. kimi* microbiota. b) Four orders belong to phylum Actinobacteria, two orders belong to phylum Proteobacteria, and one order belong to phylum Bacteroidetes were exclusively found in soil grown adult *A. kimi~~.~~*
